# A biopsychosocial & culturally-anchored suicide postvention model for the Philippines

**DOI:** 10.3389/fpsyt.2026.1772957

**Published:** 2026-02-18

**Authors:** Evangeline Bascara dela Fuente, Rowalt Alibudbud

**Affiliations:** 1 Department of Psychiatry and Behavioral Medicine, College of Medicine, University of the Philippines – Manila, Manila, Philippines; 2 National Teachers Training Center for the Health Professions, University of the Philippines – Manila, Manila, Philippines; 3 Department of Sociology and Behavioral Sciences, College of Liberal Arts, De La Salle University, Manila, Philippines

**Keywords:** bereavement, biopsychosocial model, cultural adaptation, mental health equity, Philippines, postvention, suicide

## 1 Introduction

The suicide of a loved one causes complex grief, trauma, stigma, and long-term mental-health risks for survivors. Many need specialized support beyond general bereavement services, but postvention remains underdeveloped in many low- and middle-income countries (LMICs) (1, 2).

In the Philippines, recent trends in deaths by suicide highlight the need for coordinated postvention. The WHO estimated age-standardized suicide mortality rate was about 3.42 per 100,000 in 2019. The 2018 Mental Health Act signifies meaningful policy progress (3). Yet, only 5% of the total public health expenditure has been historically allocated to mental health, and the number of mental health professionals remains low (4–6). Additionally, inequities in service access continue to exist, especially among socially marginalized groups, such as informal-sector workers, residents of rural *barangays* (villages), students, indigenous peoples, and LGBTQ+ communities (7–9).

## 2 A biopsychosocial & culturally-anchored model

The biopsychosocial model emphasizes that the expression of distress arises from the interaction among biological, psychological, and social perspectives (10).

Biologically, survivors of suicide loss may experience traumatic stress responses, such as disrupted sleep, somatic symptoms, and neuroendocrine activation. In the psychological domain, grief, guilt, shame, meaning-making, risk of complicated grief, and posttraumatic growth must be considered (2, 11, 12). Social factors include religious and spiritual frameworks, family and community ties, cultural stigma surrounding suicide, and resource limitations within health systems (13–15).

In the Philippine context, the extended family, church and pastoral networks, barangay units, and peer groups play essential roles (14, 15). Tackling stigma, facilitating communal healing, and providing network-based care are vital (14). Additionally, Filipino cultural concepts such as *kapwa* (shared self), *bayanihan* (community solidarity), *hiya* (shame), and strong faith traditions (e.g., Catholic, Muslim, Indigenous) shape meaning and coping strategies (11, 13–17).

A postvention model must comprehensively address biopsychosocial factors (2, 12, 18), as well as relevant issues like limited mental health staff, decentralized health systems, stigma, health literacy gaps, and resource shortage (4–6, 8). Commonly marginalized groups (e.g., informal workers, indigenous communities, urban slums must be given due consideration as well.

This paper presents a postvention model tailored to the Philippine context. Although informed by established frameworks such as the TAPS model, it is distinguished by its integration of Filipino psychological and cultural concepts and its explicit responsiveness to structural constraints, including limited mental health resources and workforce shortages usually seen in LMICs (4–6, 19–21). Unlike postvention models developed in high-income and Western settings, which often assume specialist-driven and individual-focused care (19–21), the proposed model emphasizes community-based support, collective coping, and feasible strategies for resource-limited contexts.

## 3 A postvention model for the Philippine context

In designing a postvention model for the Philippine context, evolving needs can be addressed through several phases, with attention to timelines (19–21). Comprehensive postvention interventions must target the biopsychosocial domains across each phase and require the availability of a crisis response team. Crisis response teams are groups of trained individuals in community settings who are well-versed in legal matters, communications/public relations, and mental health (21).

To enhance the implementation of these postvention crisis response teams, more explicit guidance on how local government units (LGUs) and barangays can operationalize the proposed phases within constrained budgets may be provided. For instance, postvention teams may be supported through modest allocations from the mandated barangay development fund (e.g., earmarking a proportion of the government-mandated 20% development fund for health-related postvention activities), prioritizing low-cost strategies such as task-sharing, basic psychosocial skills training, and referral coordination. In settings where no psychiatrist is available, its implementation may rely on trained barangay health workers, teachers, or faith leaders, with supervision and technical support from specialists and professionals provided by regional or national offices of the Department of Health.

### 3.1 Phase 1: crisis containment (24–72 hours)

This phase targets the immediate post-loss period, which is within 24–72 hours of a suicide and involves focusing on safety, reducing distress, connecting survivors with support, and laying the groundwork for grief (20).

To address the biological needs, there is a need for a Barangay Health Worker (BHW) or peer-mentor to visit the immediate social circle within 24–72 hours of a suicide, offering psychoeducation on trauma responses like sleep issues, appetite changes, and panic (2, 12, 18). To address psychological needs, trained BHWs, religious leaders, and volunteers in Psychological First Aid (PFA) will need to conduct supportive visits to normalize responses and identify high-risk symptoms such as self-harm, guilt, and suicidal thoughts (22).

For social and cultural needs, community members, local leaders, and peer groups will need to facilitate bayanihan—neighbors, church groups, and volunteers supporting one another with meals, errands, and companionship—to reduce isolation (23, 24). Faith leaders can offer compassionate pastoral visits framing suicide as a result of psychological distress rather than sin, and promote familiar practices like storytelling, meals, and prayer vigils, which can reinforce kapwa and ease grief (23, 25, 26).

### 3.2 Phase 2: stabilization (day 4 to 3 months)

This phase aims to engage survivors in deliberate grief work, facilitate meaning-making, promote integration of the death into their life narrative, ensure connection with support systems, and initiate the process of rebuilding (20). Low-cost group sessions led by trained lay counselors, supervised by mental health professionals, can address biological needs during this phase. The sessions should include relaxation techniques, stress management methods (e.g., deep breathing, yoga, tai chi), nutrition, and sleep hygiene, recognizing that bodily distress often triggers emotional dysregulation (27, 28). To meet psychological needs, conducting culturally adapted grief and trauma groups in local dialects, such as Taglish or Cebuano, using storytelling and expressive arts like song, collage, and drama can be helpful (29). Moreover, to process grief, survivors can also re-author their stories and attend peer-led groups with survivors who have experienced personal growth (30).

To address social and cultural needs, it is necessary to facilitate regular survivor-peer groups at barangay health centers or church halls. Family members can also be trained to support the survivor, understand grief, and reduce stigma. Collaborate with school and LGU programs for youth survivors. Using familiar community rituals (e.g., pamisa) can serve as opportunities for meaning-making and social reintegration (23, 25, 26). Emphasize the utang na loob (debt of gratitude) narrative, in which survivors may later give back as peer mentors, transforming their loss into acts of helping others (14, 31).

### 3.3 Phase 3: post-traumatic growth and integration (3 months and beyond)

This stage supports survivors in finding renewed meaning in life, reintegrating into the community, and experiencing post-traumatic growth, rather than just returning to their previous level of functioning (20). To meet biological needs, survivors showing signs of complicated grief, depression, or suicidal thoughts are referred through barangay health networks to psychiatrists or social workers. Meanwhile, the larger group can be encouraged to join physical or community activities that promote wellness, such as exercise groups. To address psychological needs in this phase, encourage survivors to become peer mentors, lead new groups, and share their stories publicly if they are willing (29). Facilitating “life after loss” workshops that support post-traumatic growth can also be beneficial (32).

To meet social and cultural needs during this stage, it may be helpful to institutionalize survivor-led outreach programs, including school talks, barangay resilience training, and church-based groups. This promotes bayanihan and kapwa-oriented community healing (13, 16, 17). Recognition events, such as local awards, can also help reduce stigma and shift survivors’ roles to helpers. Additionally, rituals of ongoing connection, such as memorials and masses, can transform the legacy of loss into one of service (33). Faith communities can also be engaged to craft narratives of hope, purpose, and redemption, while avoiding moralizing interpretations.

## 4 Discussion

### 4.1 Health and social equity considerations

The adaptation must prioritize equity, focusing on marginalized Filipinos in rural, urban, or indigenous settings facing barriers like poverty, stigma, and isolation (9, 34). It must not be elite-centric; instead, use barangay-based, culturally sensitive, peer-led structures. Due to stigma linked to religious beliefs, family shame, and media sensationalism (14, 35), postvention should include psychoeducation and community dialogue to shift blame. Indigenous survivors may have different views on death and mourning (36), requiring involvement of translators, faith leaders, and elders. Given limited mental health funding, few psychiatrists, and psychiatric beds (4–6, 37), the approach must focus on training non-specialists, task-shifting, and community networks to reach marginalized groups. Additionally, the lack of a national suicide registry and limited epidemiological data (38) necessitate research that includes these populations and culturally specific grief processes (4, 5).

### 4.2 Implementation challenges

Several challenges can impede the adaptation and implementation of suicide postvention efforts. First, due to limitations in the Philippine mental health system and human resources (4), the approach must emphasize task-sharing with lay and peer providers. Second, in many Filipino communities, suicide is often linked to sin, family shame, or supernatural causes. As a result, faith leaders may unintentionally reinforce stigma (14). Training is essential to foster culturally competent, non-judgmental responses.

Third, the Philippines’ health system is highly decentralized, with LGUs managing barangay and municipal health services, which complicates the creation of uniform standards and continuity (39). Therefore, coordination among barangays, church networks, NGOs, and academia is necessary. Fourth, relying on external funding may not be sustainable. As a result, integrating services into barangay health systems and securing LGU budgets for postvention are critical.

Fifth, outcome measures should extend beyond metrics like reduced suicidal ideation to include culturally grounded markers of recovery, including kapwa-based belonging, strengthened community ties, and shared growth narratives following loss. To enhance rigor and support future evaluative work, the framework may point to potential proxy indicators of these outcomes, such as perceived mutual support, participation in communal rituals or support activities, and collective meaning-making processes. Future research can further develop and validate these culturally informed indicators to assess community-level healing and recovery within the Philippine postvention context. Lastly, indigenous groups, LGBTQ+ communities, migrants, and urban poor populations require culturally tailored modules. Language diversity, literacy levels, and local belief systems must all be considered (8, 36).

### 4.3 Recommendations for policy, training & research

To improve suicide postvention implementation, policies should ensure sustainability and scalability. LGUs should pass ordinances that integrate bereavement postvention into barangay health plans, in line with the Mental Health Act. At least 5% of mental health funds should go to postvention services. A national registry should track survivors, loss circumstances, and service use (38). Expanding the number of trained laypersons and peer mentors, and embedding the model in barangay health networks rather than relying on tertiary clinics, are essential to mitigate the shortage of the mental health workforce.

The implementation of suicide postvention should be rooted in ongoing training to ensure program continuity amid limited mental health professionals in the Philippines. A tiered training model is vital, including PFA for BHWs and faith leaders, grief support for peer mentors, and advanced specialist training. These programs should also include cultural competency modules covering Filipino values, indigenous mourning, minority stress in LGBTQ+ survivors, and economic marginalization (8, 36). Support can come from hospital residencies, university practicums, and internships where psychiatric residents, psychology, and social work students support community postvention under supervision.

To improve the suicide postvention program, it must be continuously monitored and updated to meet changing needs (38). Conducting mixed-methods studies among suicide-loss survivors in Philippine settings like rural barangays, urban slums, and indigenous communities is essential to identify variations and needs. The postvention strategies should be regularly assessed for process, outcome, and equity metrics, such as engagement, grief distress, posttraumatic growth, peer-mentor activation, and access across social groups. Culturally adapted tools for measuring posttraumatic growth, communal healing, and *kapwa*-based belonging can also be developed.
